# Hierarchical Bayesian spatial models for alcohol availability, drug "hot spots" and violent crime

**DOI:** 10.1186/1476-072X-5-54

**Published:** 2006-12-07

**Authors:** Li Zhu, Dennis M Gorman, Scott Horel

**Affiliations:** 1 Department of Epidemiology and Biostatistics, School of Rural Public Health, Texas A&M Health Science Center, College Station, TX77843-1266, USA

## Abstract

**Background:**

Ecologic studies have shown a relationship between alcohol outlet densities, illicit drug use and violence. The present study examined this relationship in the City of Houston, Texas, using a sample of 439 census tracts. Neighborhood sociostructural covariates, alcohol outlet density, drug crime density and violent crime data were collected for the year 2000, and analyzed using hierarchical Bayesian models. Model selection was accomplished by applying the Deviance Information Criterion.

**Results:**

The counts of violent crime in each census tract were modelled as having a conditional Poisson distribution. Four neighbourhood explanatory variables were identified using principal component analysis. The best fitted model was selected as the one considering both unstructured and spatial dependence random effects. The results showed that drug-law violation explained a greater amount of variance in violent crime rates than alcohol outlet densities. The relative risk for drug-law violation was 2.49 and that for alcohol outlet density was 1.16. Of the neighbourhood sociostructural covariates, males of age 15 to 24 showed an effect on violence, with a 16% decrease in relative risk for each increase the size of its standard deviation. Both unstructured heterogeneity random effect and spatial dependence need to be included in the model.

**Conclusion:**

The analysis presented suggests that activity around illicit drug markets is more strongly associated with violent crime than is alcohol outlet density. Unique among the ecological studies in this field, the present study not only shows the direction and magnitude of impact of neighbourhood sociostructural covariates as well as alcohol and illicit drug activities in a neighbourhood, it also reveals the importance of applying hierarchical Bayesian models in this research field as both spatial dependence and heterogeneity random effects need to be considered simultaneously.

## Background

With the ability to properly account for high variance of estimates in small geographic areas and clarify overall geographic trends and patterns, Bayesian methods are becoming popular tools for disease mapping. Besag *et al*. [1] described a Bayesian approach which separated spatial effects from heterogeneity. Waller *et al*. [2] developed a Bayesian hierarchical model that accommodated covariates and spatial structure which evolved over time. The widespread use of geographic information systems (GIS) and links to statistical packages have further encouraged spatial data analysis [3-5]. With the development of Markov Chain Monte Carlo (MCMC) methods and software such as WinBUGS [6,7], Bayesian approaches are being applied to the analysis of many social and health problems in addition to disease mapping and modelling. In particular, Berry *et al*. [8], Cohen *et al*. [9] and Law and Haining [10] used Bayesian statistics for analyzing crime data. Random effects modeling is often used to deal with the problem of overdispersion in modeling count data [11,12]. Poisson models require that mean and variance are equal, while in real data, overdispersion often results in larger variance than mean.

This paper reports the results of fitting random effect models to census tract-level high violence areas for the City of Houston, Texas, using WinBUGS and its spatial analysis extension GeoBUGS[13] for model fitting. Model fit comparison is accomplished using the deviance information criterion [14]. These analyses will inform the growing literature within public health and criminology that is focused on the geospatial relationship between aspects of the physical and social environment and the commission of violent crime [15-18].

The remainder of this paper is organized as follows. The Method section describes the datasets pertaining to alcohol availability, drug hot spots, violent crime and sociodemographic characteristics at the census-tract level in Houston, Texas. It also undertakes an analysis of these datasets in which the alcohol and the drug covariates are included with neighborhood sociostructural variables using four random effect models, and the models are compared based on Deviance Information Criterion. The Result section reports the results of the chosen Bayesian model. Map decomposition is applied to interpret the distribution of high-violence areas in Houston. The Discussion section provides a comparison of the findings to previous studies and presents some possible avenues for future research. The Conclusion section summarizes the major findings in this research.

## Methods

### Data

The sample for this study comprised of 439 census tracts from Houston for which violent crime data were reported by the city police department. Houston is the largest city in Texas and the fourth largest in the USA. The boundaries for the tracts were those established for the 2000 US Census. Census tracts are not necessarily corresponding to neighborhoods in a socially meaningful sense, but they are considered to be the most appropriate boundary to use in assessing the relationship between neighborhood structure and violent crime [19,20], because they are relatively homogeneous with regard to population and are the best local areas where the required data are available. Three archival datasets were employed in the present study. The first one included monthly police reports of four violent crime categories of murder, rape, robbery and aggravated assault in 2000, extracted from the City of Houston Police Department website [21]. The violent crime data are based on first reports of offenses (that is, before investigation and final classification of crimes). The website also contained monthly reports of narcotic drug-law violations. Like the violent crime data, the drug-law violation data were also based on first reports of offenses. Available evidence suggests that police data represent a valid indicator of actual drug activity in a community [22]. Such "call for assistance" data have been used in previous studies of alcohol availability and violent crime, and have strengths as well as limitations relative to official crime records [23-25]. Close to 98% of the data were successfully geocoded and aggregated to census tract level in the analysis. The second dataset pertained to alcohol outlets. A list of all alcohol outlets active in the year 2000 in Houston was obtained from the website of the Texas Alcoholic Beverage Commission (TABC) [26]. There were a total of 6,609 alcohol outlets in the dataset, each of which included the name, geographic location, and type of permit or license of the outlet. Almost all of the outlets (99.5%) were successfully geocoded by street address using Centrus Desktop [27]. TABC classified the outlets by type of consumption allowed, specifically, off-premise versus on-premise. 1,480 (22.4%) of all outlets were on-premise, 3,094 (46.8%) off- premise, and 2,035 (30.8%) combined on-/off- premise. Outlet density was defined as number of outlets per 100 persons in a census tract. Principal component analysis identified total alcohol outlet (including on-, off-, and on-/off- premise) density to be the major factor in this group of covariates.

The third dataset used in the study pertained to 12 neighborhood sociostructural factors. These variables, which were grouped under three broad categories, were selected as they had been used in previous ecologic studies of alcohol availability and crime [16,25,28]. We use these variables as potential covariates in our analysis to keep in line with the existing literature and for possible comparisons between different studies. We realized that there might be collinearity issue and our study area might have a unique set of covariates which may not be exactly the same as previous studies, so we conducted a variable reduction using principal component analysis described below. The data were extracted from Summary File 1 and Summary File 3 of the 2000 US Census [29]. Of these variables, six were measures of concentrated disadvantage (% families below poverty line, % families receiving public assistance, % unemployed individuals in civilian workforce, % female-headed households with children, % Black, and % Latino), three were measures of residential instability (% of residents over age 5 who have lived in the same house for 5 or more years, % homes that are owner-occupied, and % vacant housing units), and three were sociodemographic measures of the resident population (adult to child ratio, population density, and % population that is male and aged 15–24). Since these variables were highly correlated with each other [30], principal component analysis was conducted to reduce the number of covariates. To retain the basic component structure of these neighbourhood sociostructural factors, principal component analysis was done separately for a) the six variables of concentrated disadvantage, b) the 3 measures of residential instability, and c) the three sociodemographic measures. In each of the three groups, components with eigenvalues greater than 1 were extracted and the variable with the highest factor loading for each extracted components was selected. Four variables were selected from the three groups, which were a) % of families below poverty, and % of Latino from the concentrated disadvantage measures, b) % owner-occupied housing units from the measure of residential instability, and c) % population that is male and aged 15–24 from the socidemographic measures.

### Bayesian hierarchical modelling

The Bayesian approach has become standard in the epidemiology and environmental health literature, but has only recently been used in sociological applications. It is called "hierarchical" because it uses multiple levels of analysis in an iterative way. Unlike the conventional statistical inference which derives the average estimates of parameters, hierarchical Bayesian modelling produces parameter estimates for each individual analysis unit by borrowing information from all analysis units, the customary Bayesian "borrow of strength" effect. In a standard Poisson model, the variance is required to be equal to the mean. But in reality many Poisson models have more variances and these are called over-dispersed Poisson models. Hierarchical Bayesian modelling identifies these "extra variances". In the case of spatial statistics in non-Bayesian approach, if there is high uncertainty in the regression model, a regression that explains only a small amount of variance is obtained. In a hierarchical Bayesian model, on the other hand, the "unexplained variance" is usually identified as either spatially-correlated effects or heterogeneity effects. The ability to incorporate prior knowledge without the restriction of classical distributional assumptions makes Bayesian inference a potent forecasting tool in a wide variety of fields.

In the first stage of the Bayesian hierarchical model, we specified a likelihood model for the vector of observed crime counts given the vector of relative risks of crimes, and then specified a prior model over the space of possible relative risks at the second stage. Using software packages such as WinBUGS/GeoBUGS [31,13] or sophisticated computation algorithms could yield a set of posterior means for the relative risks given the observed crime counts. The set of posterior means or medians of the relative risks was then used to create a map to visualize the high- or low-risk census tracts. Crude maps were developed from the likelihood model (the first stage) only, and often feature large outlying relative risks in small areas (where the population is small). Hence, crude maps usually show high uncertainty. They also fail to catch similarity of relative risks in nearby or adjacent regions. An appropriately-tailored Bayesian approach will incorporate spatial assumptions and achieve spatial smoothing by borrowing information from all individuals.

The violent crime counts could be modelled as a conditional Binomial(*n, p*) variable with the population sizes to be *n *and a unknown rate *p*. An initial check into the data reveals that the population sizes in the majority of the census tracts are large (in thousands) and the violent crimes are rare, with an average rate of around 5%. So we use the Poisson approximation to binomial distribution at the first stage of likelihood specification. The model assumes that the number of crime counts in region *i, Y*_*i*_, has a conditional independent Poisson distribution with mean *E*_*i *_exp(*μ*_*i*_). Here *E*_*i *_is the expected number of events, which is fixed and proportional to the corresponding known population *n*_*i*_. Specifically, we set *E*_*i *_= *Rn*_*i*_, where the proportional constant *R *is the grand rate (i.e., the total number of events divided by the total population). Hence exp(*μ*_*i*_) is the relative risk: regions with exp(*μ*_*i*_) > 1 generally have greater numbers of observed incidence than expected, and vice versa for regions with exp(*μ*_*i*_) < 1. Thus *μ*_*i *_is log-relative risk, modelled linearly as

*μ*_*i *_= **x**'_*i*_**β **+ *θ*_*i *_+ *φ*_*i*_, *i *= *1*, ..., *I *    (1)

Here **x**'_*i *_are region-specific covariates, and **β **is a vector of fixed effects. *θ*_*i *_and *φ*_*i *_are the region-specific random effects capturing heterogeneity and spatial dependence, respectively. The typical way to impose this structure is to assume that *θ*_*i*_'s are i.i.d. Gaussian variables with mean *0 *and variance 1/*τ *and *φ*_*i*_|*φ*_*j*≠*i *_~ *N*(μφi
 MathType@MTEF@5@5@+=feaafiart1ev1aaatCvAUfKttLearuWrP9MDH5MBPbIqV92AaeXatLxBI9gBaebbnrfifHhDYfgasaacH8akY=wiFfYdH8Gipec8Eeeu0xXdbba9frFj0=OqFfea0dXdd9vqai=hGuQ8kuc9pgc9s8qqaq=dirpe0xb9q8qiLsFr0=vr0=vr0dc8meaabaqaciaacaGaaeqabaqabeGadaaakeaaiiGacqWF8oqBdaWgaaWcbaGae8NXdy2aaSbaaWqaaiabdMgaPbqabaaaleqaaaaa@31DC@, σφi2
 MathType@MTEF@5@5@+=feaafiart1ev1aaatCvAUfKttLearuWrP9MDH5MBPbIqV92AaeXatLxBI9gBaebbnrfifHhDYfgasaacH8akY=wiFfYdH8Gipec8Eeeu0xXdbba9frFj0=OqFfea0dXdd9vqai=hGuQ8kuc9pgc9s8qqaq=dirpe0xb9q8qiLsFr0=vr0=vr0dc8meaabaqaciaacaGaaeqabaqabeGadaaakeaaiiGacqWFdpWCdaqhaaWcbaGae8NXdy2aaSbaaWqaaiabdMgaPbqabaaaleaacqaIYaGmaaaaaa@32DC@), *i *= *1*,...,*I*, where

μφi=∑j≠iwijφij∑j≠iwij and σφi2=1λ∑j≠iwij.
 MathType@MTEF@5@5@+=feaafiart1ev1aaatCvAUfKttLearuWrP9MDH5MBPbIqV92AaeXatLxBI9gBaebbnrfifHhDYfgasaacH8akY=wiFfYdH8Gipec8Eeeu0xXdbba9frFj0=OqFfea0dXdd9vqai=hGuQ8kuc9pgc9s8qqaq=dirpe0xb9q8qiLsFr0=vr0=vr0dc8meaabaqaciaacaGaaeqabaqabeGadaaakeaaiiGacqWF8oqBdaWgaaWcbaGae8NXdy2aaSbaaWqaaiabdMgaPbqabaaaleqaaOGaeyypa0ZaaSaaaeaadaaeqbqaaiabdEha3naaBaaaleaacqWGPbqAcqWGQbGAaeqaaOGae8NXdy2aaSbaaSqaaiabdMgaPjabdQgaQbqabaaabaGaemOAaOMaeyiyIKRaemyAaKgabeqdcqGHris5aaGcbaWaaabuaeaacqWG3bWDdaWgaaWcbaGaemyAaKMaemOAaOgabeaaaeaacqWGQbGAcqGHGjsUcqWGPbqAaeqaniabggHiLdaaaOGaeeiiaaIaeeyyaeMaeeOBa4MaeeizaqMaeeiiaaIae83Wdm3aa0baaSqaaiab=z8aMnaaBaaameaacqWGPbqAaeqaaaWcbaGaeGOmaidaaOGaeyypa0ZaaSaaaeaacqaIXaqmaeaacqWF7oaBdaaeqbqaaiabdEha3naaBaaaleaacqWGPbqAcqWGQbGAaeqaaaqaaiabdQgaQjabgcMi5kabdMgaPbqab0GaeyyeIuoaaaGccqGGUaGlaaa@68E2@

Weights *w*_*ij *_are fixed constants. With this structure, *θ*_*i*_'s capture heterogeneity among regions and *φ*_*i*_'s capture spatial dependence or autocorrelation. In practice, a common choice is to let *w*_*ij *_= *0 *unless areas *i *and *j *are adjacent, in which case *w*_*ij *_= *1*. Other forms of *w*_*ij *_(often using distance between centroids of regions *i *and *j*) are possible [32,33]. This distribution for **ϕ **= (*φ*_1_,..., *φ*_*I*_) is called an intrinsic conditionally autoregressive specification [2,10,13], which for brevity we typically write in vector notation as **ϕ **~ ICAR(*λ*). A fully Bayesian model specification is completed by adding prior distributions on **β**, *τ *and *λ*. Without prior expectations about direction and magnitude of the covariate effects, a vague but proper prior distribution is put on the regression coefficients **β**. Prior distribution for *τ *and *λ *is *Gamma(a, b) *with mean *a*/*b *and variance *a*/*b*^2^. With no prior estimation for precisions of the random effects, small values of *a *and *b *are chosen to assume large variance. Here we have chosen *Gamma(0.001,0.001)*, a vague prior for precision parameters of both effects.

Principal component analysis was conducted to obtain a subset of four neighborhood variables. The main effects of interest in this study are those of alcohol outlet densities and drug-law violation densities on violence. So the covariate matrix includes six explanatory variables, and the dependant variable is the occurrence of violence in each census tract which follows a Poisson distribution as specified earlier. We fitted four models with or without random effects, and assessed the performance of them using Bayesian approaches. Model I contained the fixed effects only. It assumes that the parameters were fixed for each region *i *and all relevant covariates had been correctly specified. An extension was to specify random effects which included either one or both of unstructured (**θ**) and spatial dependence effects (**ϕ**). These were called Model II (fixed effects and **θ**), Model III (fixed effects and **ϕ**), and Model IV (full model), respectively.

Statisticians have long paid attention to the model selection problem. For years, Bayesian statisticians were advised to use only Bayes factors [34] for this purpose. However, Bayes factor becomes quite difficult both to compute and interpret for high-dimensional hierarchical models, and is not well-defined for models having improper prior distributions. The difficulties with Bayes factor have led to a host of alternative model choice criteria. Most recently, Spiegelhater *et al*. [14] proposed a generalization of the Akaike Information Criterion [35] that was based on the posterior distribution of the deviance statistic

*D*(ϑ) = -2log *p*(**y|θ**) + 2log *f*(**y**).

Here *p*(**y|θ**) is the likelihood function for the observed data vector **y **given the parameter vector **θ**, and *f*(**y**) is a standardizing function of the data alone. In this approach the model *fit *is summarized by the posterior expectation of the deviance, D¯
 MathType@MTEF@5@5@+=feaafiart1ev1aaatCvAUfKttLearuWrP9MDH5MBPbIqV92AaeXatLxBI9gBaebbnrfifHhDYfgasaacH8akY=wiFfYdH8Gipec8Eeeu0xXdbba9frFj0=OqFfea0dXdd9vqai=hGuQ8kuc9pgc9s8qqaq=dirpe0xb9q8qiLsFr0=vr0=vr0dc8meaabaqaciaacaGaaeqabaqabeGadaaakeaacuWGebargaqeaaaa@2DD5@ = *E*_*θ*|*y*_(*D*), while model *complexity *is captured by the number of effective parameters *p*_*D*_, which is defined as expected deviance minus deviance evaluated at the posterior expectations, i.e.

*p*_*D *_= *E*_*θ*|*y*_(*D*) - *D*(*E*_*θ*|*y*_(*θ*)) = D¯
 MathType@MTEF@5@5@+=feaafiart1ev1aaatCvAUfKttLearuWrP9MDH5MBPbIqV92AaeXatLxBI9gBaebbnrfifHhDYfgasaacH8akY=wiFfYdH8Gipec8Eeeu0xXdbba9frFj0=OqFfea0dXdd9vqai=hGuQ8kuc9pgc9s8qqaq=dirpe0xb9q8qiLsFr0=vr0=vr0dc8meaabaqaciaacaGaaeqabaqabeGadaaakeaacuWGebargaqeaaaa@2DD5@ - *D *(θ¯
 MathType@MTEF@5@5@+=feaafiart1ev1aaatCvAUfKttLearuWrP9MDH5MBPbIqV92AaeXatLxBI9gBaebbnrfifHhDYfgasaacH8akY=wiFfYdH8Gipec8Eeeu0xXdbba9frFj0=OqFfea0dXdd9vqai=hGuQ8kuc9pgc9s8qqaq=dirpe0xb9q8qiLsFr0=vr0=vr0dc8meaabaqaciaacaGaaeqabaqabeGadaaakeaaiiGacuWF4oqCgaqeaaaa@2E81@)

*Deviance information criterion *(DIC) is then defined as the summation of *fit *and *complexity*, i.e.

DIC = D¯
 MathType@MTEF@5@5@+=feaafiart1ev1aaatCvAUfKttLearuWrP9MDH5MBPbIqV92AaeXatLxBI9gBaebbnrfifHhDYfgasaacH8akY=wiFfYdH8Gipec8Eeeu0xXdbba9frFj0=OqFfea0dXdd9vqai=hGuQ8kuc9pgc9s8qqaq=dirpe0xb9q8qiLsFr0=vr0=vr0dc8meaabaqaciaacaGaaeqabaqabeGadaaakeaacuWGebargaqeaaaa@2DD5@ + *p*_*D *_= 2D¯
 MathType@MTEF@5@5@+=feaafiart1ev1aaatCvAUfKttLearuWrP9MDH5MBPbIqV92AaeXatLxBI9gBaebbnrfifHhDYfgasaacH8akY=wiFfYdH8Gipec8Eeeu0xXdbba9frFj0=OqFfea0dXdd9vqai=hGuQ8kuc9pgc9s8qqaq=dirpe0xb9q8qiLsFr0=vr0=vr0dc8meaabaqaciaacaGaaeqabaqabeGadaaakeaacuWGebargaqeaaaa@2DD5@ - *D*(θ¯
 MathType@MTEF@5@5@+=feaafiart1ev1aaatCvAUfKttLearuWrP9MDH5MBPbIqV92AaeXatLxBI9gBaebbnrfifHhDYfgasaacH8akY=wiFfYdH8Gipec8Eeeu0xXdbba9frFj0=OqFfea0dXdd9vqai=hGuQ8kuc9pgc9s8qqaq=dirpe0xb9q8qiLsFr0=vr0=vr0dc8meaabaqaciaacaGaaeqabaqabeGadaaakeaaiiGacuWF4oqCgaqeaaaa@2E81@)

Smaller values of DIC indicate a better-fitting model. Table 1 lists deviance summaries for the four models. A comparison of DIC shows that the mixed effect models (Models II, III, and IV) are much better than the fixed effect model (Model I). The number of *effective *parameters in Model I is the number of covariates, while adding random effects for each of the 439 census tracts in Models II through IV makes the number of *effective *parameters far less than the total number of model parameters due to "borrowing of strength" across individual-level parameters in hierarchical models. Model II contributes 357 extra parameters, and Model III (with stronger restriction of spatial dependence) contributes 351 extra parameters to Model I. Model IV has a slightly smaller DIC value than Models II and III, and a fraction of extra effective parameter compared with Model III. The comparison of the three random effects models suggests that Model IV may be regarded as more parsimonious, yet the significance to the apparent difference of 10 between Models III and IV is hard to assess [36]. Here we consider another criterion

**Table 1:** Deviance summaries for the four hierarchical models

Model	D¯ MathType@MTEF@5@5@+=feaafiart1ev1aaatCvAUfKttLearuWrP9MDH5MBPbIqV92AaeXatLxBI9gBaebbnrfifHhDYfgasaacH8akY=wiFfYdH8Gipec8Eeeu0xXdbba9frFj0=OqFfea0dXdd9vqai=hGuQ8kuc9pgc9s8qqaq=dirpe0xb9q8qiLsFr0=vr0=vr0dc8meaabaqaciaacaGaaeqabaqabeGadaaakeaacuWGebargaqeaaaa@2DD5@	*D*(ϑ¯ MathType@MTEF@5@5@+=feaafiart1ev1aaatCvAUfKttLearuWrP9MDH5MBPbIqV92AaeXatLxBI9gBaebbnrfifHhDYfgasaacH8akY=wiFfYdH8Gipec8Eeeu0xXdbba9frFj0=OqFfea0dXdd9vqai=hGuQ8kuc9pgc9s8qqaq=dirpe0xb9q8qiLsFr0=vr0=vr0dc8meaabaqaciaacaGaaeqabaqabeGadaaakeaaiiGacuWFrpGsgaqeaaaa@2E73@)	*p*_*D*_	DIC
I Fixed effects only	6081.78	6072.78	9.004	6090.78
II Fixed and heterogeneity	2784.25	2417.84	366.41	3150.66
III Fixed and dependence	2786.43	2425.65	360.78	3147.21
IV Full model	2776.66	2415.75	360.91	3137.57

α=sd(φ)sd(φ)+sd(θ),
 MathType@MTEF@5@5@+=feaafiart1ev1aaatCvAUfKttLearuWrP9MDH5MBPbIqV92AaeXatLxBI9gBaebbnrfifHhDYfgasaacH8akY=wiFfYdH8Gipec8Eeeu0xXdbba9frFj0=OqFfea0dXdd9vqai=hGuQ8kuc9pgc9s8qqaq=dirpe0xb9q8qiLsFr0=vr0=vr0dc8meaabaqaciaacaGaaeqabaqabeGadaaakeaaiiGacqWFXoqycqGH9aqpdaWcaaqaaiabdohaZjabdsgaKjabcIcaOiab=z8aMjabcMcaPaqaaiabdohaZjabdsgaKjabcIcaOiab=z8aMjabcMcaPiabgUcaRiabdohaZjabdsgaKjabcIcaOiab=H7aXjabcMcaPaaacqGGSaalaaa@4399@

where *sd(.) *is empirical marginal standard deviation. Hence *α *is the proportion of variability in the random effects that is due to spatial dependence. Larger values (near 1) suggest a dominating spatial dependence, while smaller values (near 0) suggest a negligible one. Recall that we specified the same *gamma *prior distribution for *τ *and *λ*, i.e., *α *= 1/2. Fitting data with the full model obtains the posterior distribution of *α *with mean 0.584, median 0.582, and a 95% credible interval (0.450, 0.733). This indicates that approximately 60% of excess variability is due to spatial dependence, while 40% is due to unstructured random noise. This confirms that Model IV (full model with both spatial dependence and spatial heterogeneity) is the best among the candidate models.

Two parallel sampling chains were run with overdispersed initial values. Convergence was assessed by checking the trace plots of the samples, autocorrelation functions, the Gelman-Rubin convergence statistic [37], and Monte Carlo standard errors [31]. The four models described above had different "burn-in" (pre-convergence) periods, with slower convergence for the more complex models. Convergence was detected at 20,000 iterations in the full model. For each model, the first 20,000 pre-convergence samples were discarded and each chain was run for a further 25,000 iterations, giving 50,000 samples with acceptable Monte Carlo errors (<5% of the posterior standard deviation).

Sensitivity analysis was conducted to investigate whether results in the analysis remained essentially unchanged in the presence of different prior information. Vague but proper prior distributions *Normal(0, .000001) *and *Gamma (0.001,0.001) *[31] were first specified for the covariate coefficients and precision parameters, respectively. Then we made modifications to the prior distributions, recomputed the posterior quantities of interest and checked whether they imposed a practical impact on interpretations or decisions. We adapted an informative prior distribution *Gamma (0.1, 0.1) *and another commonly used *Gamma (0.5, 0.0005) *[38] for this purpose. The three distributions gave almost identical results for all the parameters, indicating the results are robust to changes in prior information.

## Results

The covariate values were first transformed by natural logarithm and then standardized (centered around mean and divided by standard deviation) to speed convergence [5]. Table 2 reports the results of Model IV from the 50,000 samples after burn-in period. Summarized are posterior statistics for the covariate regression coefficients (*β *'s), and the precision parameters for spatial (*φ *'s) and unstructured (*θ *'s) random effects. Listed in the table are posterior mean, standard deviation, Monte Carlo error, median, 2.5^th ^percentile, 97.5^th ^percentile (with the last two items being simply the 95% credible interval), and relative risk (corresponding to one standard deviation change in the covariate) which is computed as the exponential of posterior median. For the regression coefficients, the sign (positive or negative) and the size of the parameters indicate the direction and magnitude of the fixed effects. Intercept can be interpreted as the log risk of crime over the entire city when all the covariates are taken to be zero (i.e. equal to their means). This value could not be assessed without this analysis. In this study, Houston shows a risk of exp(-0.314) = 0.73. Of the four neighborhood covariates, only males of age 15 to 24 show an effect on violence, and this is the only neighbourhood covariate included in the final model. The neighbourhood covariate shows a 16% decrease in relative risk for each increase the size of its standard deviation. The main effects of interest are both significant, with coefficients of 0.151 and 0.914, respectively.

**Table 2:** Posterior summaries for regression and precision coefficients

Parameter	Mean	Standard Deviation	Monte Carlo error	2.5%	Median	97.5%	Relative Risk
intercept	-0.314	0.018	0.0003	-0.351	-0.314	-0.279	----
% Population of male aged 15–24	-0.174	0.034	0.0013	-0.242	-0.173	-0.108	0.841
Alcohol outlet density	0.152	0.032	0.0009	0.091	0.151	0.214	1.163
Drug-law violation density	0.913	0.038	0.0023	0.832	0.914	0.986	2.494
Unstructured random effect	10.63	5.387	0.258	5.489	9.181	24.89	----
Spatial dependence	2.067	0.867	0.041	1.046	1.870	4.246	----

While data transformation makes interpretation somewhat difficult, it is instructive to examine in detail a typical (and real) inner-city census tract with a population the size of 1191, 12 alcohol outlets, and 116 drug-law violation reports in the year 2000. Assuming all other covariates remain the same, if the number of alcohol outlets increases to 48 (with a standard deviation of 36), then, on average, the relative risk of violence will increase 16%; if the number of drug-law violation reports increases to 168 (with a standard deviation of 52), then the relative risk of violence increases 1.5 times. It is apparent that the effects of alcohol outlets are quite modest compared to the effects of drug-law violations. It is hard to imagine any real-word community would increase the alcohol outlets such dramatically, but increase of drug-law violations from 116 to 168 would be possible. Precision for spatial random effects is lower than the unstructured heterogeneity. Later analysis of the maps shows the same tendency.

**Figure 1:**
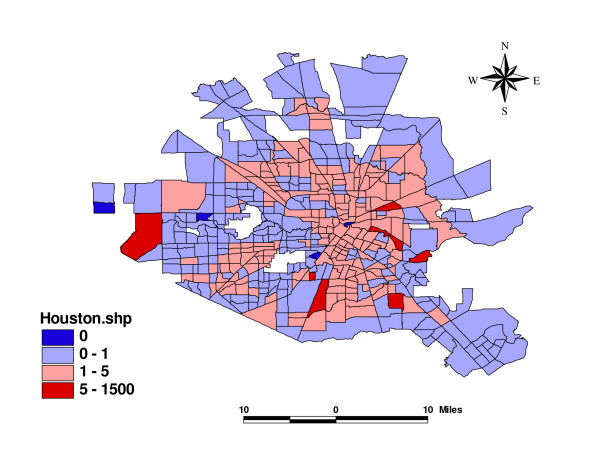
Standardized Incidence Ratio of Violence in Houston, Texas.

Figure 1 plots the standard incidence ratio (SIR) of violence in the study area. Standard incidence ratio is the crude ratio of observed violence counts to expected counts. As described in the Method section, expected counts are thought of as fixed and proportional to the known population. No covariate or random effect is considered in their calculation. The pivotal value 1 means that observed and expected counts are the same. Areas with SIR>1 have larger observed violence counts than expected. The map includes blank areas with missing data, as those areas belong to other cities (Bellarie, Jacinto City, etc.). Generally, the northern and outskirt areas of the city have lower violence incidence, and the inner city has higher violence risk. The numbers in parenthesis are counts of census tracts in each level. About 55% (240 of 439) of all the census tracts have SIR lower than 1, while extreme values do exist. Four of the small census tracts have observed violence counts 10 times higher than the expected, while four very small areas have SIR = 0.

**Figure 2:**
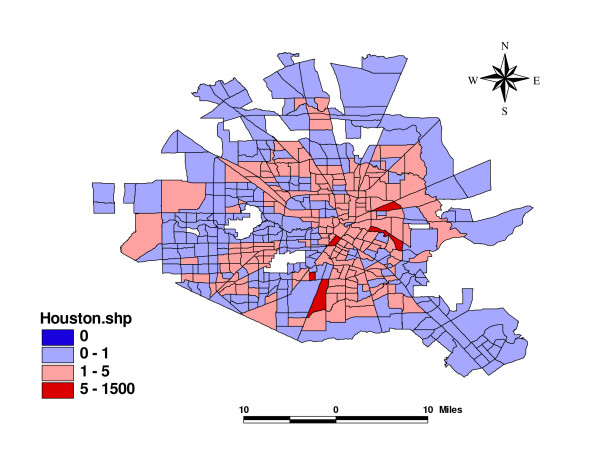
Fitted Incidence Ratio of Violence in Houston, Texas.

Figure 2 presents the map of the fitted incidence ratio of violence in the study area. Fitted incidence ratio is SIR multiplied by exponential of *μ*_*i*_, which is modelled in (1). This index accounts for information on all three aspects of the fixed effects, the unstructured heterogeneity random effect, and the spatial dependence effect. The figure clearly shows characteristic Bayesian shrinkage of the crude rate toward the local average rate. In particular, no census tract is now assigned a value of exactly zero, and the extremely high rate in one of the small areas (east of the city) has been substantially reduced. The high values in the inner city remain high. There also appears to be some tendency for local clustering of similar values, the probable outcome of the *ICAR*(*λ*) portion in the model.

**Figure 3:**
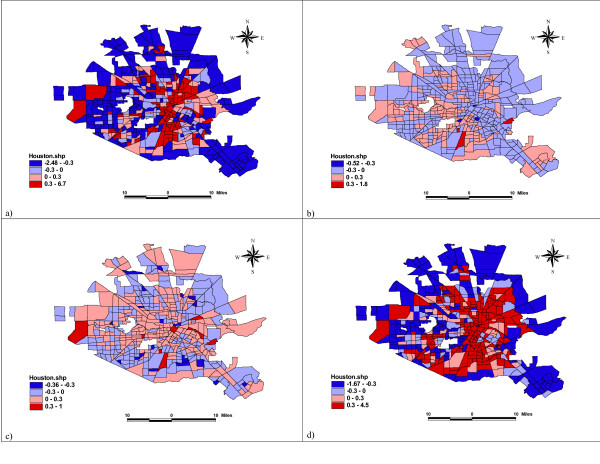
Map Decomposition of the log Relative Risks for a) Fixed Effect, b) Neighborhood Sociostructural Covariates, c) Alcohol Outlet Densities, and d) Illicit Drug-Law Violations. Four levels (from the lightest to the darkest) shown in the maps are <= -0.3, -0.3 – 0, 0 – 0.3, and >0.3.

**Figure 4:**
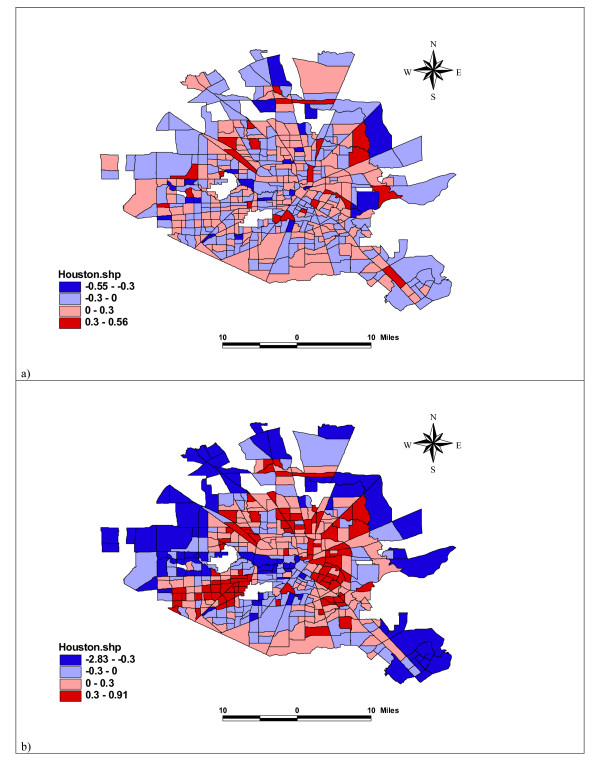
Map Decomposition of the log Relative Risks for the Random Effect Part into a) Unstructured, and b) Spatially Dependence Random Effects. Four levels (from the lightest to the darkest) shown in the maps are <= -0.3, -0.3 – 0, 0 – 0.3, and >0.3.

Law and Haining [10] developed a map decomposition strategy that provided some interesting insights into different factors in Bayesian spatial modelling. Here we adapt the method and pay special attention to the visualization of the results in our model. The log relative risk *μ*_*i *_is a random variable which consists of three parts: the deterministic fixed effect **x**'_*i*_**β**, the spatial dependence random effect *φ*_*i*_, and the unstructured heterogeneity effect *θ*_*i*_. Figure 3 shows the maps of the posterior means of **x**'_*i*_**β **for all covariates, the neighborhood covariates, the alcohol factor, and the drug covariate, respectively. It reveals that illicit drug-law violation is the major contributing factor, with highly spatially varied values dominating the distribution pattern of the fixed effects from all covariates. The distributions of the neighborhood sociostructural factors and of alcohol density are more or less more uniform. Figure 4 shows the maps of the random effects in log-relative risk, calculated using the posterior means of the unstructured heterogeneity effect *θ*_*i*_, and the spatial dependence random effect. Neither of the random effects is dominating over the other, though the spatial dependence effect is slightly higher than the unstructured effect. The variability observed in the spatial dependence effects is higher, and this confirms the result about *α*, the proportion of the variability in the random effects that is due to spatial dependence. The posterior mean of *α *= 58% indicates that neither of the random effects plays a more important role than the other. The map of the spatial effects shows a clear pattern of local clustering of similar values, while it is difficult to detect a distributional pattern in the unstructured heterogeneity effect.

**Figure 5:**
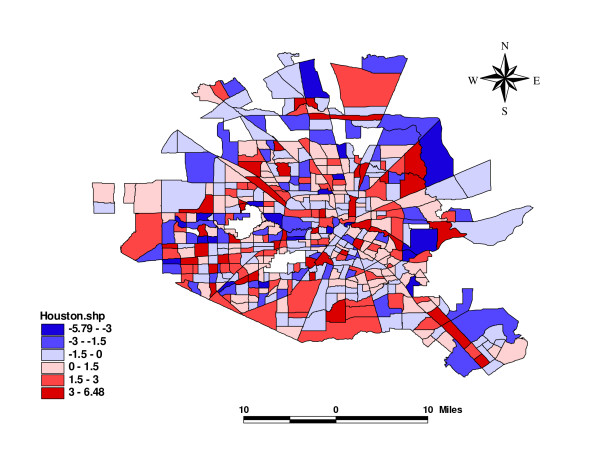
Residuals for the Bayesian Hierarchical Model.

Finally, the map of the posterior means of residuals (Figure 5) is used to assess the overall model goodness-of-fit. Residual is the difference between observed and fitted values of the dependent variable, the counts of violence in this case. If the model fits well and all relevant covariates are included, spatially independent residuals are expected. A visual check on the map suggests that there is no spatial distributional pattern in the residuals. There could still be important independent variables that are missing in the model, but they probably do not have significant spatial structure and hence do not have an impact on the residual map.

## Discussion

The analysis presented suggests that activity around illicit drug markets is more strongly associated with violent crime than is alcohol outlet density. This supports previous studies that have shown a spatial link between drug "hot spots" and violence [39]. However, it is possible that some of the association between drug crime density and violent crime rates found in the present study is due to the fact that the data used to assess each of these came from a common source, namely the Houston police department. So, while available evidence suggests that police data provide a meaningful indicator of drug activity in a community [22], it would have been preferable to have some additional measure of this variable that was independent of law enforcement records (e.g., neighborhood surveys). It also needs to be noted here that an association has been identified between violent crimes and neighborhood covariates as well as alcohol and drug activity, it requires further investigation as to whether the exposure (alcohol and drug activity) and the outcome (violence) are both consequences of the same unmeasured phenomenon. There is not necessarily a causal relation between the exposure and the outcome.

A major contribution of the current study is to reveal the importance of spatial analysis in the research into the interaction of alcohol availability, illicit drug market, and violent crime. The Bayesian hierarchical modelling approach provides the methodology that incorporates complex data and model levels, with spatial dependence structure. Freely available software such as WinBUGS/GeoBUGS enables wider use of the developed methods. A useful extension to the methods described here would be to assess spatially heterogeneous interactions between disparate data sources [40]. In the current study, the impact of alcohol availability and illicit drug activity on violent crime is assumed to be constant over the whole study area. There are theoretical reasons to believe that the associations may be different in different geographic locations. The challenge for ecological research programs is to develop models of the spatial interactions of people and places to predict violence across community areas.

## Conclusion

In this paper we have presented a three-level Bayesian hierarchical modelling approach to model the occurrence of violence in Houston, Texas. The first level was the likelihood of the occurrence of violence that follows a Poisson distribution. Level 2 modelled the log relative risk as a linear combination of three components which accounted for fixed effects of possible covariates, random effects of unstructured heterogeneity and spatial dependence. At level 3, non-informative hyperprior distributions were assigned to the precision parameters for the random effects. Eight explanatory variables were identified via principal component analysis. Four models that included/excluded the two random terms were compared based on Deviance Information Criterion and proportion of the variability in the random effects that is due to spatial autocorrelation. The full model which considered both unstructured and spatial dependence random effects was selected as the best fitted model. Sensitivity analysis was performed to check whether the prior assumptions on the precision parameters had an undue effect on the results. Of the three fixed effects that contributed to the relative risk of violent crime, drug-law violation explained a greater amount of variance in violent crime rates than alcohol outlet densities and neighbourhood sociostructural variables. The census tract level random effects included both a spatial dependence and an unstructured heterogeneity effect, suggesting that it is necessary to carry out this research in a Bayesian hierarchical framework where different formats of random effects and fixed effects are considered. The residual map indicated that the quality of model fit was satisfactory.

## Competing interests

The author(s) declare that they have no competing interests.

## Authors' contributions

LZ contributed to manuscript writing, literature review, study design, and statistical analysis. DMG made substantial contributions to conception, design of the study and acquisition of data. SH contributed to data acquisition, data development and cartography.
